# Red cell size factor is a sensitive index in the early diagnosis of nondigestive tract cancer-related anemia: An observational study

**DOI:** 10.1097/MD.0000000000039736

**Published:** 2024-09-27

**Authors:** Bicui Zhan, Yongjia Zhu, Jiahong Yu, Qiaojuan Zhu, Huaying Zhang, Xiaoqiang Ye

**Affiliations:** a Department of Laboratory Medicine, Hangzhou TCM Hospital Affiliated to Zhejiang Chinese Medical University, Hangzhou, Zhejiang, China; b Department of Laboratory Medicine, The Second Haining People’s Hospital, Haining, Zhejiang, China.

**Keywords:** early diagnosis, nondigestive tract cancer-related anemia, red cell size factor, risk

## Abstract

Cancer-related anemia (CRA) is a common comorbidity in cancer patients, and it can lead to a worse prognosis. The aim of this cross-sectional study is to investigate the clinical value of the red cell size factor (Rsf) in the early diagnosis of nondigestive tract CRA. A total of 231 patients with nondigestive tract solid cancer were included, and they were divided into anemic and nonanemic subjects according to the hemoglobin (Hb) levels. A BC-7500 blood analyzer was used to detect the indices of red blood cell and reticulocyte, and the mean corpuscular volume (MCV), mean reticulocyte volume (MRV), reticulocyte hemoglobin (RHE) content, and reticulocyte production index were observed. Subsequently, the Rsf was calculated. Receiver operating characteristic curve analysis was used to evaluate the identifying power of Rsf for anemia diagnosed by the combination of RHE and reticulocyte production index. The adjusted-multivariate analysis and quartiles were used to assess the relation of reduced Rsf level with the risk and incidence of anemia diagnosed by combining the MCV, MCH, and mean corpuscular hemoglobin concentration (MCHC), respectively. Rsf levels showed no statistical differences between anemia and nonanemia subjects grouped by Hb (*P* > .05). Rsf has a high correlation with the RHE level (*R* > 0.900, *P* < .001), or MCV, mean corpuscular hemoglobin (MCH), and MCHC in anemia patients (r: 0.435–0.802, *P* < .001). Receiver operating characteristic curves showed that Rsf had the highest overall area under curve of 0.886 (95% confidence interval: 0.845–0.927) in identifying anemia of cancer patients (*P* < .001). When the optimal cutoff values of Rsf were set at 97.05 fl in males and 94.95 fl in females, the sensitivity and specificity were 0.94 and 0.76, and 0.98 and 0.75, respectively. Being treated as a categorical variable, Rsf had a highest odds ratio value of 30.626 (12.552–74.726; *P* < .001) for the risk of anemia. The increment of Rsf quartiles was highly associated with the decreased incidence of overall anemia (*P* trend < 0.001). The study suggests that decreased Rsf level is a potentially powerful predictor of overt anemia in nondigestive tract cancer, and it can be used as a convenient, practical, cost-free, and sensitive index in early diagnosis of nondigestive tract CRA.

## 1. Introduction

Cancer is a common and important comorbidity in cancer patients at diagnosis, and it has become one of the most important causes of mortality worldwide.^[1]^ Chronic anemia is the common manifestation during the progression of cancers, which is defined as cancer-related anemia (CRA).^[2]^ Studies have reported that decreased hemoglobin levels can be found in 20–30% of cancer patients before starting treatment, and in 60–70% during therapy.^[3]^ The associations of anemia with poor prognosis of cancer patients have been clearly proved, and there is a negative impact of anemia on cancer progression, risk of death, and final survival,^[4–6]^ which means anemia is a prognostic factor for cancer patients. Therefore, it will be important for the prognostic improvement of cancer patients to early screen and diagnose CRA.

Up to now, it has been well recognized that CRA is caused by the following mechanisms: (1) nutritional deficiency, (2) ineffective erythropoiesis caused by tumor cells, (3) hemolysis, or (4) blood loss.^[7–9]^ In the etiology of CRA, the detailed mechanisms can be tacked including the decreasing of red blood cell (RBC) production, increase of RBC destruction, and loss of RBC (bleeding).^[10]^ Cancer-related low-grade chronic inflammation is the primary cause of the variety of mechanisms of CRA, which can exhibit the similar characteristics to chronic inflammation-related anemia caused by other chronic diseases.^[2]^ Some crossing mechanisms are involved in the systemic inflammation associated with CRA, and the most important ones include the inhibition of erythroid precursors, reduced erythropoietin production, shortened lifespan of erythrocyte, and the abnormality of iron metabolism.^[2,11–13]^ Previous reports have revealed that CRA is a normocytic and normochromic anemia,^[11]^ and this type of anemia is mainly characterized by the normal or low serum iron levels, a low reticulocyte count, and the abnormalities of other mature and immature RBC indices.^[14,15]^ Therefore, some parameters of mature RBC and reticulocytes associated with the etiology of CRA can be used as the early indices of identification and diagnosis in CRA.

It is defined as anemia when hemoglobin (Hb) levels in peripheral blood are below 120 g/L for women and 130 g/L for men according to the criteria of the World Health Organization.^[16]^ However, the actual criteria of anemia diagnosis differ because of the differences in ethnicity and region. Although Hb level has been used as the major diagnostic index of all types of anemia all the time, decreased Hb level simply suggests an overt anemia, which indicates Hb will have a low sensitivity to diagnose anemia in the early stage of its development. Torino et al^[17]^ revealed that mature erythrocyte and reticulocyte indices are good parameters in identifying absolute iron deficiency in anemic patients with chronic disease. Moreover, several reticulocyte maturity indices, such as medium and high fluorescence reticulocyte, immature reticulocyte fraction, reticulocyte production index (RPI), and reticulocyte hemoglobin (RHE) content are more powerful indices in early diagnosis of anemia and in the evaluation of bone marrow recovery.^[18–21]^ Recently, the red cell size factor (Rsf) joins together the volume of the mature erythrocytes and reticulocytes and has been focused on the assessment of erythropoiesis status.^[22]^ Osta et al^[23]^ reported that Rsf showed a sensitivity of 92% and a specificity of 81% in the diagnosis of iron deficiency anemia. Mean corpuscular volume (MCV) reflects the erythrocyte size and can diagnose anemia as microcytic, normocytic, or macrocytic anemia, and it is a good index in differential diagnosis of anemia.^[24]^ The specific utility of reticulocyte volume measurement has been reported as a valuable index in screening and differentiating hereditary spherocytosis,^[25,26]^ and diabetes mellitus patients can also exhibit different levels of reticulocyte volume.^[27]^ Moreover, a previous study also revealed that MRV is a valuable index in early diagnosis of CRA.^[28]^ However, the utility of Rsf calculated with MCV and MRV in CRA was rarely reported, and the actual clinical significance remains unclear. As we know, digestive tract-related cancers are usually associated with underlying chronic bleeding, and they can cause chronic iron-deficient anemia, which the laboratory characteristics differ from that of CRA itself. Therefore, the present study was undertaken to study the significance of Rsf in early identifying anemia in patients with a variety of nondigestive tract cancers.

## 2. Materials and methods

### 2.1. Sample size

In this study, the minimal sample size was calculated as follows: $n=\frac{u^{2}\sigma^{2}}{\delta^{2}}$ (*n*: sample size; *σ*: standard deviation; *δ*: allowable error; *u*: *U* value under *ɑ*=0.05). The allowable error (*δ*) was calculated as 2.8 and 2.08 g/L using the allowable CV% of 2.0%. According to the Chinese adult reference intervals for blood cell analysis,^[29]^ the total standard deviation (*σ*) is 9.2 and 8.7 g/L for males and females, respectively. Thus, the mean standard deviation (*σ*) is 9.0 g/L. Therefore, we used the *U* value (1.96), *δ*, and *σ* to calculate the minimal sample size as 72 and 40 for anemic and nonanemic subjects, respectively, in this study. Moreover, we also set the percentage of missing cases as 10%, and the final minimal sample size was 79 and 44 for males and females, respectively.

### 2.2. Study subjects

A total of 231 consecutive patients with the first confirmed diagnosis of nondigestive tract solid cancer were enrolled in this cross-sectional study. The subjects were recruited from the Hangzhou Hospital of Traditional Chinese Medicine between August 2023 and February 2024. The patients included 136 males and 95 females, aged 14–95 years. They included 95 nonanemic and 136 anemic patients. The type of cancers in the two groups was presented in Table 1. The diagnostic criteria of anemia were according to Hb < 130 g/L for males and < 115 g/L for females from the Chinese reference intervals of blood cell analysis for adults.^[29]^ The inclusion criteria were as follows: untreated solid cancer from nondigestive tract. The exclusion criteria were as follows: thrombotic diseases; primary liver and kidney disorders; a recent history of surgery; cardiovascular and cerebrovascular diseases; acute infection; and hematological and hematopoietic diseases. Because this study data were retrospectively reviewed, the informed consent of all individual participants was waived. The study was approved by the ethical committee of Hangzhou Hospital of Traditional Chinese Medicine (approval no. 2024LL068).

**Table 1:** The distribution of malignancies in cancer patients with anemia and nonanemia

Type of cancers	n	Group anemia (n)	Group nonanemia (n)	Percentage of anemia (%)
Total cancers	231	135	96	58.4
Lung cancer	30	23	7	76.7
Cholangiocarcinoma	17	13	4	76.5
Liver malignancy	17	12	5	70.6
Pancreatic malignancy	16	10	6	62.5
Prostate malignancy	18	9	9	50.0
Breast malignancy	16	8	8	50.0
Thyroid malignancy	20	8	12	40.0
Renal cancer	13	7	6	53.8
Ureteral malignancy	6	6	0	100
Cervical malignancy	9	6	3	66.7
Brain malignancy	15	6	9	40.0
Endometria malignancy	12	6	6	50.0
Osteocarcinoma	10	5	5	50.0
Bladder malignancy	9	4	5	44.4
Retroperitoneal malignancy	8	4	4	50.0
Ovary malignancy	6	5	1	71.4
Nasopharyngeal carcinoma	5	2	3	40.0
Lumbar malignancy	1	1	0	100
Parotid gland malignancy	2	0	3	0.0

### 2.3. Laboratory measurements

Blood samples were collected and treated as previously described.^[28]^ In brief, the peripheral blood of the patients was collected in vaccutainer tubes with EDTA-K_2_, sodium citrate, and anticoagulants-free (BD Inc, USA), respectively, after an overnight fast before treatment. The samples with EDTA-K2 were analyzed by using the Mindray BC-7500CRP blood cell analyzer (Mindray Inc, China), and the observed indices included the detected and calculated ones of mature RBCs and reticulocytes, immature reticulocyte indices, white blood cells, and platelet count. The Rsf was calculated according to the formula: $\sqrt{MCV*MRV}$. (MCV: mean corpuscular volume; MRV: mean reticulocyte volume). Subsequently, plasma was isolated from the specimens with sodium citrate by centrifugation at 1500 × *g* at room temperature for 10 minutes for plasma fibrinogen measurement by using the CN-9000 coagulation analyzer (Sysmex, Japan), and the sera were also obtained from the centrifugated specimens without anticoagulants for the measurements of serum albumin, creatinine (sCre), thyroid-stimulating hormone by the AU5800 biochemistry analyzer (Beckman Coulter, USA).

### 2.4. Statistical analysis

In this study, the normality of the data was analyzed by the nonparameter test (Kolmogorov–Smirnov test) initially, and normal and non-normal distributing data were presented as ±SD and median (*P*_25_–*P*_75_), respectively. Subsequently, Student *t* test and Mann–Whitney *U* test were, respectively, used to analyze the differences between the groups of normality and non-normality distribution, and Chi-square test was used to analyze the categorical variables including percentage. At the same time, the receiver operating characteristic (ROC) curves were constructed, and the area under curve was calculated, and the sensitivity and specificity of the variables were also obtained based on the optimal cutoff values in identifying the anemic and nonanemic patients. Moreover, the correlation of Rsf with the levels of mature RBC and reticulocyte indices was analyzed by the Spearman method. Finally, an adjusted-multivariate analysis was carried out by including some confounders to calculate the odds ratios (ORs) and 95% confidence intervals (95% CI) for early nondigestive tract CRA. The SPSS 25.0 software (SPSS, IBM Corp, USA) was used, and *P* < .05 was considered statistically significant.

## 3. Results

### 3.1. Basic characteristics of the cancer patients

First, the basic physiological and laboratory characteristics of the patients were investigated. In this study, there were 18 and 17 types of cancers in anemic and nonanemic patients, respectively. Although the composition percentage of the malignancies differed between the anemic and nonanemic groups, the total percentage of the top four malignancies (lung cancer, cholangiocarcinoma, liver malignancy, and pancreatic malignancy) in this study showed no statistical differences between the two groups (23/30, 77%; 13/17, 77%; 12/17, 71%; and 10/16, 63%; *P* > .05) (Table 1). Anemic patients had a higher mean age at diagnosis than that of nonanemic ones (62.08 ± 15.77 vs 53.8 ± 15.9, *P* < .001), but males and females constituted approximately the same proportion (males: 51% vs 44%, *P* > .05). However, there was no significant differences for RET#, RHE, MRV, and Rsf based on reticulocyte analyses between the two groups (*P* > .05), but other reticulocyte indices showed significantly different in the two groups (*P* < .001). The detailed results are listed in **Table 2**.

**Table 2:** Comparisons of basic characteristics and laboratory indices between anemic and nonanaemic patients with cancer

Indices	Cancer without anemia (n = 95)	Cancer with anemia (n = 136)	Statistical value	*P*
Male (n, %)	35 (36.8)	69 (50.7)	4.362	0.037
Age (yrs)	53.8 ± 15.9	62.08 ± 15.77	3.937	<0.001
BMI (kg/m^2^)	23.60 ± 3.59	22.29 ± 3.48	2.647	0.009
SBP (mm Hg)	127.6 ± 20.33	128.5 ± 20.3	0.327	0.744
DBP (mm Hg)	76.4 ± 10.7	74.1 ± 12.9	1.335	0.183
sCre (mmol/L)	62.0 (52.0–74.0)	65.0 (54.0–87.0)	1.022	0.307
ALB (g/L)	40.95 ± 4.10	34.84 ± 6.24	8.275	<0.001
Fbg (g/L)	3.00 (2.00–3.00)	3.00 (3.00–4.00)	3.500	<0.001
TSH (U/L)	2.0 (1.0–2.0)	4.02 (0–13.7)	2.166	0.030
WBC (×10^9^/L)	6.00 (5.00–7.00)	5.00 (4.00–7.00)	1.599	0.054
PLT (×10^9^/L)	209 (172–265)	207 (141–273)	0.977	0.328
Ret# (×10^9^/L)	73 (57–94)	64 (52–81)	1.888	0.059
HFR (%)	0.0 (0.0–1.0)	1.5 (0.0–3.0)	6.100	<0.001
MFR (%)	8.0 (6.0–10.0)	11.0 (8.0–14.0)	5.552	<0.001
LFR (%)	92.0 (89.0–93.0)	87.0 (83.0–97.0)	5.718	<0.001
IRF (%)	8.0 (7.0–11.0)	13.0 (9.0–17.0)	5.759	<0.001
RPI	1.0 (1.0–2.0)	0.9 (0.35–1.43)	5.450	<0.001
RHE (pg)	29.0 (28.0–30.0)	28.5 (26.0–30.0)	1.069	0.285
MRV (fL)	101.0 (97.0–104.0)	100.0 (93.3–106.8)	0.835	0.404
RSF (fL)	95.8 (92.4–98.4)	95.3 (90.0–99.5)	0.699	0.484

Data were presented as mean ± SD, median (*P*_25_–*P*_75_), or percentage.

ALB = albumin, BMI = body mass index, Cre = creatinine, DBP = diastolic blood pressure, Fbg = fibrinogen, HFR = high fluorescence reticulocyte, IRF = immature reticulocyte fraction, LFR = low fluorescence reticulocyte, MFR = medium fluorescence reticulocyte, MRV = mean reticulocyte volume, PLT, platelet, RET# = reticulocyte count, RHE = reticulocyte hemoglobin content, RPI = reticulocyte production index, Rsf = red cell size factor, SBP = systolic blood pressure, TSH = thyroid-stimulating hormone, WBC = white blood cells.

*P* values were calculated by Student *t* test, Mann–Whitney *U* test, and Chi-square test, respectively.

### 3.2. Correlation of Rsf levels with the mature RBC and reticulocyte indices

The Spearman analysis showed a negative correlation of Rsf levels with that of RBC and red cell distribution width-coefficient of variation (*P* < .01 or 0.001 in anemic patients and *P* < .05 in nonanemic ones). Rsf was positively correlated with that of RHE level (*R* = 0.927 and 0.909, *P* < .001 respectively) in the two groups, and it was also highly associated with the levels of MCH, MCV, and MCHC in anemic patients (*r*: 0.435–0.802; *P* < .001) and with that of MCV and MCH in nonanemic ones (*r*: 0.767–0.886; *P* < .001), respectively (**Table 3**).

**Table 3:** Correlation of RSF levels with the RBC and reticulocyte indices in cancer patients

Grouping	r/p	RHE	RPI	RET#	RBC	HGB	MCV	MCH	MCHC	RDW-CV
Anemic patients	r	0.927	0.317	0.171	−0.334	0.306	0.782	0.802	0.435	−0.276
	p	<0.001	<0.001	<0.05	<0.001	<0.001	<0.001	<0.001	<0.001	<0.01
Nonanemic patients	r	0.909	0.334	0.367	−0.220	0.100	0.886	0.767	−0.076	−0.226
	p	<0.001	<0.01	<0.001	<0.05	>0.05	<0.001	<0.001	>0.05	<0.05

MCH = mean corpuscular hemoglobin, MCHC = mean corpuscular hemoglobin concentration, MCV = mean corpuscular volume, RBC = red blood cell, RDW-CV = red cell distribution width-coefficient of variation, RET# = reticulocyte count, RHE = reticulocyte hemoglobin content, RPI = reticulocyte production index, Rsf = red cell size factor.

*P* values were calculated by Spearman analysis.

### 3.3. ROC curves of Rsf in identifying anemia and nonanemia classified by RHE and RPI

**Figure 1.**
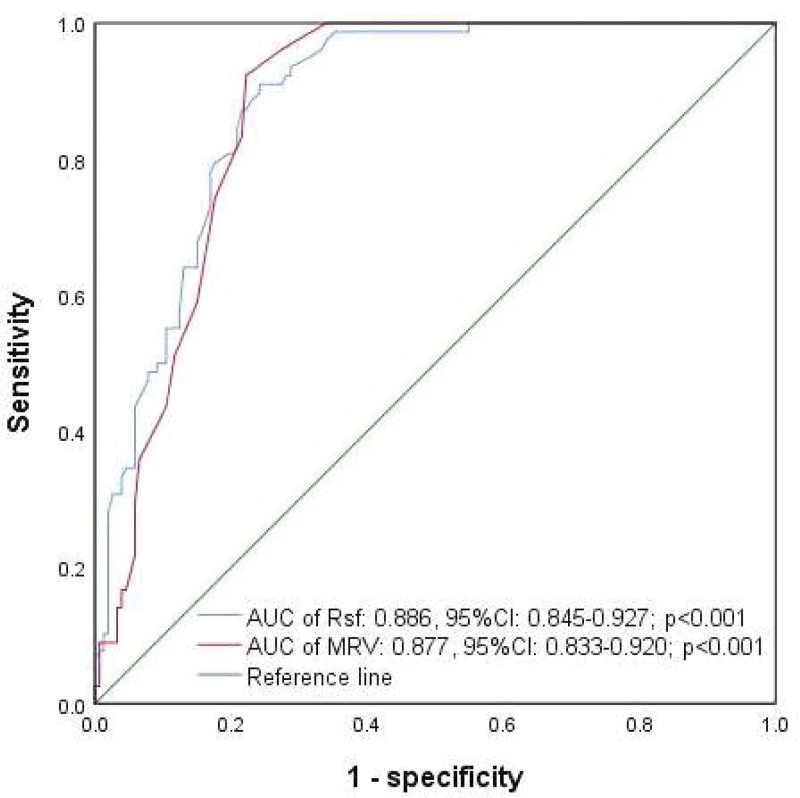
ROC curve analysis of Rsf and MRV in identifying anemia and nonanemia. AUC = area under curve, CI = confidence interval, MRV = mean reticulocyte volume, ROC = receiver operating characteristic, Rsf = red cell size factor.

Because of the highest correlation of Rsf level with that of RHE, the patients were classified as anemic and nonanemic according to the single or simultaneous decrease in RHE and RPI levels compared with their median. The mature RBC and reticulocyte indices were included to be analyzed. ROC curves analysis revealed a highest area under curve of 0.903 (95% CI: 0.848–0.959) in males and a second AUC of 0.889 (95% CI: 0.832–0.946) in females for Rsf in identifying anemia from nonanemia (*P* < .001). However, the AUC of other indices ranged from 0.466 to 0.802 in males and 0.363 to 0.843 in females. According to the optimal cutoff values of Rsf (97.05fl in males and 94.95fl in females), the sensitivity and specificity were calculated as 0.94 and 0.76, and 0.98 and 0.75, respectively (Table 4). However, when we included the data of the male and female patients to construct the ROC curve, the AUC of Rsf was higher than that of MRV(0.886, 95% CI: 0.845–0.927 vs 0.877, 95% CI: 0.833–0.920) (Fig. 1).

**Table 4:** ROC curve analysis of RSF for identifying anemia diagnosed by the combination of RHE and RPI

Variables	AUC	95%CI		*P* value	Optimal cutoff values	Sensitivity	Specificity	Youden index
		Lower	Upper					
Males								
RSF	0.903	0.848	0.959	<0.001	97.05	0.94	0.76	0.70
MRV	0.872	0.807	0.937	<0.001	101.5	1.00	0.73	0.73
MCV	0.802	0.715	0.889	<0.001	91.50	0.77	0.71	0.49
MCH	0.766	0.665	0.867	<0.001	31.50	0.63	0.79	0.41
RET#	0.814	0.732	0.897	<0.001	0.09	0.63	0.87	0.50
HGB	0.692	0.591	0.794	0.001	118.5	0.80	0.54	0.34
HCT	0.687	0.584	0.789	0.002	33.50	0.86	0.49	0.34
RBC	0.596	0.486	0.705	0.111	3.50	0.83	0.36	0.19
MCHC	0.512	0.393	0.630	0.846	321.00	1.00	0.86	0.09
RDW-CV	0.466	0.345	0.587	0.568	19.50	0.57	1.00	0.06
Females								
RSF	0.889	0.832	0.946	<0.001	94.95	0.98	0.75	0.72
MRV	0.896	0.841	0.951	<0.001	99.5	1.00	0.75	0.75
MCH	0.843	0.776	0.911	<0.001	30.50	0.93	0.68	0.61
RET#	0.810	0.734	0.911	<0.001	0.06	0.42	0.58	0.49
MCV	0.793	0.717	0.870	<0.001	89.5	0.88	0.58	0.46
MCHC	0.719	0.630	0.808	<0.001	335.50	0.91	0.49	0.40
HGB	0.684	0.589	0.779	0.001	109.50	0.79	0.48	0.27
HCT	0.656	0.557	0.755	0.004	32.50	0.77	0.48	0.25
RBC	0.480	0.374	0.586	0.713	6.50	0.23	0.99	0.01
RDW-CV	0.363	0.266	0.4591	0.012	11.0	1.00	0.00	0.00

AUC = area under curve, CI = confidence interval, HCT = hematocrit, MCH = mean corpuscular hemoglobin, MCHC = mean corpuscular hemoglobin concentration, MCV = mean corpuscular volume, MRV = mean reticulocyte volume, RBC = red blood cell, RDW-CV = red cell distribution width-coefficient of variation, RET# = reticulocyte count, RHE = reticulocyte hemoglobin content, ROC = receiver operating characteristic, RPI = reticulocyte production index, Rsf = red cell size factor.

*P* values were calculated by ROC curve analysis.

### 3.4. Levels of mature RBC and reticulocyte indices between anemic and nonanemic patients classified by MCV, MCH, and MCHC

In consideration of the highly close correlation of Rsf with MCV, MCH, and MCV in anemic patients, and the MCV, MCH, and MCV are the more sensitive and frequently-used indices of overt anemia than Hb concentrations in clinical practice, we used them to classify anemia also according to the single or simultaneous decrease in MCH, MCHC, and MCV levels compared with the optimal cutoff values calculated by the ROC curves. Under this anemia classification, the RET#, Rsf, RHE, and MRV levels showed significant differences between anemic and nonanemic groups (*P* < .05, 0.01, or 0.001) (**Table 5**).

**Table 5:** Comparisons of the various indicators levels in anemia and nonanemia grouped by MCV, MCH, and MCHC

Grouping	n	RET# (×10^12^/L)	Rsf (fl)	RHE (pg)	MRV (fl)
Nonanemia	85	0.074 (0.057–0.096)	99.2 (8.6–96.1)	30.0 (29.0–31.0)	104.0 (102.0–108.0)
Anemia	146	0.068 (0.053–0.082)	93.1 (96.5–102.2)	28.0 (26.0–29.0)	98.0 (92.0–102.3)
Statistical value		2.299	8.988	7.231	7.410
*P*-value		<0.05	<0.001	<0.001	<0.001

Data were presented as mean ± SD, median (*P*_25_–*P*_75_) or percentage.

MCH = mean corpuscular hemoglobin, MCHC = mean corpuscular hemoglobin concentration, MCV = mean corpuscular volume, MRV = mean reticulocyte volume, RET# = reticulocyte count, RHE = reticulocyte hemoglobin content, Rsf = red cell size factor.

*P* values were calculated by Mann–Whitney *U* test, respectively.

### 3.5. Adjusted-multivariate analysis of Rsf, MRV, and Hb for anemia risk

**Figure 2.**
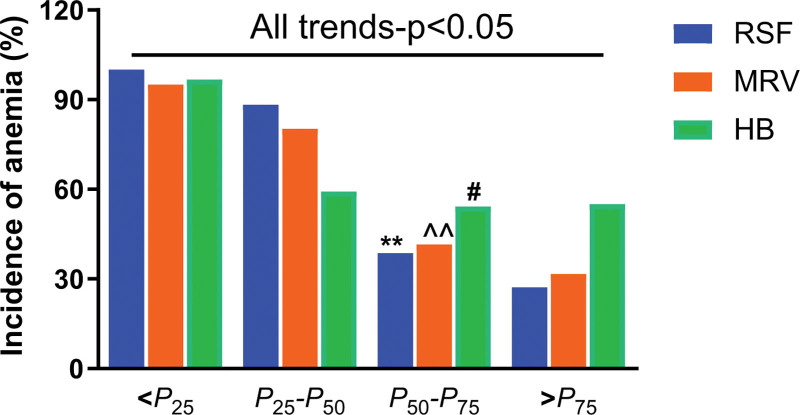
The incidence of nondigestive tract CRA in different quartiles of Rsf, MRV, and Hb levels. Hb = hemoglobin, MRV = mean reticulocyte volume, Q = quartile, Rsf = red cell size factor. Trend-p was analyzed by trend Chi-square test. Compared with Q2, #*P* < .05, **,^^*P* < .01 analyzed by Chi-square test.

According to the anemia and nonanemia grouped by MCV, MCH, and MCHC, gender, age, body mass index, and blood pressure of the patients, which could potentially influence the numbers and morphology of mature RBC and reticulocyte, were treated as the confounders and an adjusted-multivariate logistic analysis was performed. The regression analysis showed that Rsf, MRV, and Hb are all the independent risk factors for anemia, whether they were used as continuous or categorical variables (*P* < .01 or 0.001). Moreover, Rsf showed an extremely high OR value of 30.626 (12.552–74.726), which means the overall risk of anemia in patients with below the cutoff values of Rsf was approximately 31 times higher than that with above the cutoff values (**Table 6**). Moreover, when the cancer patients were divided into four groups according to the quartiles of Rsf, MRV, and Hb levels in males and females, the incidence of overall anemia showed a decreasing trend with the increasing of their quartiles, respectively (p-trend < 0.01), but the incidence based on Rsf quartiles exhibited a more significant decreasing trend (**Fig. 2).**

**Table 6:** Adjusted-multivariate regression analysis for anemia risk classified by MCV, MCH and MCHC

Variables	OR (95%CI)	*P* value
Rsf		
Continuous variable	1.477 (1.311–1.664)	<0.001
Optimal cutoff from ROC curve		
Male > 97.1 fl, female > 95.0 fl	1	
Male < 97.1 fl, female < 95.0 fl	30.626 (12.552–74.726)	<0.001
MRV		
Continuous variable	1.170 (1.110–1.232)	<0.001
Optimal cutoff from ROC curve		
Male > 101.5 fl, female > 99.5 fl	1	
Male < 101.5 fl, female < 99.5 fl	16.732 (7.381–38.110)	<0.001
Hb		
Continuous variable	1.035 (1.017–1.053)	<0.001
Optimal cutoff from ROC curve		
Male > 118.5 g/L, female > 109.5 g/L	1	
Male < 118.5 g/L, female < 109.5 g/L	2.452 (1.283-4.684)	<0.01

The results were analyzed by adjusting the confounders including sex, age, BMI, SBP, DBP when the indices were presented as continuous and categorical variables based on the cutoff values, respectively.

BMI = body mass index, CI = confidence interval, DBP = diastolic blood pressure, Hb = hemoglobin, MCH = mean corpuscular hemoglobin, MCHC = mean corpuscular hemoglobin concentration, MCV = mean corpuscular volume, MRV = mean reticulocyte volume, OR = odds ratio, Rsf = red cell size factor, SBP = systolic blood pressure.

*P* values were calculated by binary multivariate logistic regression analysis.

## 4. Discussion

In this study, the levels of mature RBC indices showed significant differences between the anemic and nonanemic group classified by Hb concentrations, and decreased levels of some reticulocyte indices also were found. However, we did not find a marked decrease in the RET count, RHE, MRV, and Rsf levels in the anemic group, which exhibited similar results to our previous study in CRA study.^[28]^ The initial results suggest that the Hb level may not be an appropriate index for early diagnosis of anemia. RHE content has been recognized as an easily applicable and early marker to assess iron deficiency,^[30,31]^ and it is a better predictive index than hemocytometric parameters such as MCV, RDW, and Hb,^[32,33]^ and also is a simple screening and diagnostic marker for ID and iron deficiency anemia.^[33,34]^ Thus, Hb is possibly not a sensitive parameter for the diagnosis of nondigestive tract CRA because some early anemic patients may demonstrate a normal Hb concentration and decreased levels of reticulocyte indices such as RHE, MRV, and Rsf the similar overall levels of RHE, MRV, and Rsf between the two Hb-classified groups have suggested that possibility. Therefore, the single use of Hb concentration in anemia diagnosis may miss some early anemic patients with cancer. Thus, as an overt feature of anemia analysis, Hb concentration is not an ideal index in the early diagnosis of nondigestive tract CRA.

In practice, neither RHE nor RPI is the commonly-used indices, but there has been enough evidence to prove them the sensitive indices in the diagnosis of early anemia. The abovementioned studies have presented the diagnostic significance of RHE in anemia. RPI can also be used to evaluate erythropoietic activity and the etiology of anemia and is a good measure of whether the reticulocyte production is adequate for the degree of anemia present.^[35,36]^ Thus, the use of combined RHE and RPI will be better for the diagnosis of nondigestive tract CRA. In this study, based on the anemia classified by the combination of RHE and RPI median, the Rsf exhibited a higher AUC than that of other RBC and reticulocyte indices in identifying anemia from nonanemia in all cancer patients. Furthermore, when the optimal cutoff values of Rsf (97.05 fL in males and 94.95fL in females) were used to identify anemia, the sensitivity in both was found at 93% and 98% with the specificity of 76% and 75%, respectively. Our accumulated evidence revealed that Rsf may be a more valuable index than other indices of RBC and reticulocyte in the identification of anemia and nonanemia, and it can be used as a powerful diagnostic index of early nondigestive tract CRA.

For a long time, RBC count, Hb concentration, and the calculated indices of RBC (MCV, MCH, and MCHC) have been the most commonly-used indices in screening anemia. MCV is a good index in screening thalassemia,^[37]^ and in differentiation of thalassemia from iron deficiency anemia^[38]^ or nonthalassemic microcytosis.^[39]^ However, although decreased RBC count and low Hb concentration have the high power to diagnose iron-deficient anemia, the mature RBC has a life span of about 120 days, and it will take a long time for Hb and RBC indices to reflect the iron deficiency. Therefore, only use of the mature RBC indices will not be suitable for the early screening of reduced hematopoiesis. MRV and MCV represent the size of the recently produced and mature RBCs, respectively, before the blood collection.^[40]^ It is known that reduced overall reticulocyte size may cause a low overall RBC size, Consequently, the patient will exhibit an early low MRV followed by a decreased MCV. Therefore, in this study, we used the Rsf from the combination of MRV and MCV to explore its diagnostic significance in nondigestive tract CRA. We found that Rsf level was highly correlated with that of RHE in either anemic or nonanemic subjects (*R* = 0.927 and 0.909, respectively), indicating almost comparability with other report,^[41]^ and further revealing the similar ability of Rsf and RHE in the diagnosis of anemia. The study also showed a high correlation of Rsf level with that of MCV and MCH (*R* = 0.767–0.886) in male and female patients, respectively, but there was a weaker correlation with RBC or Hb. The present study further implies RBC and Hb are less conducive than Rsf for the early diagnosis of nondigestive tract CRA, and it would be of importance to explore the utility of Rsf in early diagnosis of anemia.

MCV, MCH, and MCHC represent RBC size and the content and concentrations of Hb in a single RBC, respectively. It has been well known that cancer progression can influence RBC size, and the MCV is a valuable index of prognosis in cancers.^[42]^ As mentioned above, cancer progression can inhibit bone marrow hematopoiesis to lead to anemia, thus to cause abnormal reticulocyte size. Therefore, we speculated that nondigestive tract CRA can simultaneously cause the abnormality of Rsf levels. In this study, the parallel testing approach was used to divide the patients into anemic and nonanemic ones by the combination of MCV, MCH, and MCHC. Differed from that based on Hb-classified anemia and nonanemic, the levels of RET#, Rsf, MRV, and RHE showed remarkable differences between the two groups, and the anemic patients exhibited significantly decreased levels of the four indices, which further indicated that Hb concentration is a less sensitive index for the early diagnosis of overt nondigestive tract CRA. As a sensitive measured index reflecting anemia, the joint of MCV, MCH, and MCHC is better than Hb in the diagnosis of overt anemia. As we know, in laboratory assays of complete blood count, decreased hemoglobin concentration usually represents the final result of anemia, and is looked at as the apparent parameter of anemia development and progression. Thus, the early assessment of the pathological process of anemia development will be greatly important in predicting anemia. In our study, after we identified the nondigestive tract CRA by using the above combination, we performed the adjusted-multivariate regression analysis including some confounders to evaluate the associations of Rsf, Hb, and MRV with the risk of anemia. We found that all three indices exhibited high OR values calculated from the risk regression analysis of nondigestive tract CRA, indicating that, as the independent risk factors, decreased levels of these indices are highly associated with the increased risk for nondigestive tract CRA. However, Rsf has the highest OR value of 30.626 amongst them, which suggests it is a more powerful and independent predicting index for the risk of overt nondigestive tract CRA than any other one in this study.

To further reveal the relation of decreased Rsf level with the risk of nondigestive tract CRA, we investigated the incidence of anemia in different quartiles of Rsf, MRV, and Hb levels. We found a reduced incidence of anemia with the increment of the quartile levels, but a much significantly reduced incidence of CRA was along with the increase of Rsf level. As a simple and fast calculated index including MCV and MRV on Mindray BC-7500CRP analyzer, Rsf can be easily obtained, and it has been proven the powerful clinical significance in diagnosis and risk evaluation of nondigestive tract CRA. Although the previous study has revealed that MRV is an independent risk factor for anemia in overall cancer patients,^[28]^ this present study actually exhibited a far more higher OR value of Rsf than that of MRV, which suggests Rsf is a more powerful and practical index than MRV and other indices in the early diagnosis of nondigestive tract CRA. However, our present results were measured by the Mindray BC-7500CRP analyzer, and whether similar results can be produced in the early diagnosis of nondigestive tract CRA from the measurements of MRV and MCV on other analyzers, which suggests that it would be of importance to draw a definite conclusion for the use of different blood analyzers.

Within our knowledge, there is at least a limitation in this study. This study included many types of nondigestive tract cancer, and it may not be comparable for the baseline levels of MRV and MCV between different types of cancer patients, thus it possibly lead to some potentially nonsignificant differences between Rsf levels in some anemic and nonanemic patients. Therefore, further studies including the same or similar type of cancer subjects will obtain more definitive conclusions. Despite the potential limitation, the present study has indicated that Rsf level was strongly and negatively associated with the incidence of nondigestive tract CRA.

## 5. Conclusions

The present study indicates that the Rsf calculated from MCV and MRV is a sensitive, convenient, cost-free, and practical index in the early diagnosis of nondigestive tract CRA, and reduced Rsf level is the powerful and independent risk factor for overt anemia in patients with nondigestive tract cancer.

## Acknowledgments

This work was supported by the Science and Technology Project of Haining (grant no. 2023031, 2022126).

## Author contributions

**Conceptualization:** Bicui Zhan, Yongjia Zhu, Xiaoqiang Ye.

**Data curation:** Bicui Zhan, Yongjia Zhu, Huaying Zhang, Jiahong Yu, Xiaoqiang Ye.

**Formal analysis:** Bicui Zhan, Huaying Zhang, Qiaojuan Zhu.

**Investigation:** Bicui Zhan, Huaying Zhang, Jiahong Yu, Qiaojuan Zhu.

**Methodology:** Bicui Zhan, Huaying Zhang, Jiahong Yu.

**Supervision:** Bicui Zhan, Yongjia Zhu, Xiaoqiang Ye.

**Validation:** Bicui Zhan, Jiahong Yu, Qiaojuan Zhu, Xiaoqiang Ye.

**Writing—original draft:** Bicui Zhan, Xiaoqiang Ye.

**Writing—review & editing:** Bicui Zhan, Yongjia Zhu, Huaying Zhang, Jiahong Yu, Xiaoqiang Ye.

**Funding acquisition:** Yongjia Zhu.

**Resources:** Yongjia Zhu, Jiahong Yu, Qiaojuan Zhu.

**Software:** Qiaojuan Zhu.

**Project administration:** Xiaoqiang Ye.
